# Prevalence and Associated Metabolic Factors of Gallstone Disease in the Elderly Agricultural and Fishing Population of Taiwan

**DOI:** 10.1155/2014/876918

**Published:** 2014-02-23

**Authors:** Hsi-Che Shen, Yi-Chun Hu, Yu-Fen Chen, Tao-Hsin Tung

**Affiliations:** ^1^New Taipei City Hospital, Taipei 241, Taiwan; ^2^Taipei Medical University, Taipei 110, Taiwan; ^3^Department of Healthcare Management, Yuanpei University, Hsinchu 700, Taiwan; ^4^Oriental Institute of Technology, Taipei 220, Taiwan; ^5^Business Place Hygiene Management, Department of Health, Taipei City Government, Taipei 110, Taiwan; ^6^Institute of Health and Welfare Policy, National Yang-Ming University, Taipei 112, Taiwan; ^7^Department of Nursing, Kang-Ning Junior College of Medical Care and Management, Taipei 114, Taiwan; ^8^Cheng Hsin General Hospital, Shih-Pai 112, Taipei, Taiwan; ^9^Faculty of Public Health, School of Medicine, Fu-Jen Catholic University, Taipei 242, Taiwan; ^10^Department of Crime Prevention and Correction, Central Police University, Taoyuan 333, Taiwan

## Abstract

*Purpose*. To evaluate sex-related differences in the prevalence of and cardiovascular risk factors related to gallstone disease (GSD) in an elderly agricultural and fishing population of Taipei, Taiwan. *Methods*. The study sample consisted of 6511 healthy elderly participants (3971 men and 2540 women) who were voluntarily admitted to a teaching hospital for a physical checkup in 2010. The participants' blood samples and real-time ultrasound fatty liver results were collected. *Results*. The prevalence of GSD in the study population was 13.2%, which increased significantly with population age (*P* < .0001). Women were associated with significantly higher GSD prevalence than men (14.8% versus 12.2%; for the chi-square test, *P* = .003). In a multiple logistic regression analysis, female sex, older age, and metabolic syndrome (MetS) were significantly associated with GSD. Multiple logistic regression analysis also revealed that obesity (odds ratio (OR) = 1.26, 95% confidence interval (CI): 1.09–1.44) and metabolic factors (one or 2 versus none, OR = 1.48, 95% CI: 1.08–1.76) were significantly associated with GSD in women but not in men. *Conclusion*. In the study population, female sex, older age, and MetS were associated with higher GSD prevalence. The population exhibited other sex-related differences.

## 1. Introduction

Gallstone disease (GSD) is the most common disease of the biliary system and most cases are asymptomatic [1]. It is also one of the most common disorders affecting emergency room patients with abdominal discomfort, epigastric pain, nausea, vomiting, and loss of appetite [2]. GSD is typically benign, but its associated complications contribute substantially to health care costs and can even be life threatening [3]. Several large studies have evaluated the prevalence of GSD in the general population by using ultrasonography [4–7]. The pathogenesis of GSD is considered multifactorial and probably develops from complex interactions among several genetic and environmental factors [8, 9]. Clearly understanding the risk factors for GSD could facilitate the early identification of patients with GSD and might reduce the risk of GSD in some patients [1].

GSD fulfills the Wilson screening criteria, which specify that the disease is a critical health problem and are listed as follows: the disease natural history should be understood; there should be a recognizable latent or early symptomatic stage; there should be a test that is easy to perform and interpret, acceptable, accurate, reliable, sensitive, and specific; there should be an accepted treatment recognized for the disease; treatment should be more effective if started early; there should be a policy on who should be treated; diagnosis and treatment should be cost effective; and case finding should be a continual process [10]. In agricultural and fishing populations, providing appropriate training to maintain good health is a requirement because long or irregular work hours can cause adverse health effects. However, according to our research, few clinical evidence-based studies have determined the prevalence and possible etiology of GSD in the elderly agricultural and fishing population of Taiwan. Therefore, the purpose of this study was to determine the prevalence of and associated risk factors for GSD, as well as any sex-related differences in GSD prevalence that could have implications on overall GSD pathogenesis, in an elderly population. We evaluated sex-related differences in the prevalence of and cardiovascular risk factors for GSD in an elderly agricultural and fishing population by using a cohort of healthy volunteers subjected to a health screening examination in Taipei, Taiwan.

## 2. Methods

### 2.1. Data Resource and Data Collection

The sample for this cross-sectional study consisted of 6511 elderly adults (3971 men and 2540 women) with agricultural and fishing occupations who were voluntarily admitted to a teaching hospital in Northern Taiwan for an annual physical checkup between January 2010 and December 2010. Blood samples and ultrasound fatty liver results were collected. The participants completed a questionnaire on their demographic data, personal disease history, medications, and family history. Fasting blood samples were collected by clinical nurses using venipuncture. Overnight-fasting serum and plasma samples (from whole blood preserved with EDTA and NaF) were stored at −20°C until analysis. All procedures were performed according to the guidelines of the hospital institutional ethics committee and adhered to the tenets of the Declaration of Helsinki. All patient information was anonymous.

In this study, the age ranges of study participants were stratified as follows: 60–64 yrs, 65–74 yrs, 75–84 yrs, and ≥85 yrs. The medical histories and measurements of the participants were obtained by well-trained nurses. Personal and family histories of hypertension, type 2 diabetes, cardiovascular diseases, and other chronic diseases were obtained by a structured health interview questionnaire. The study participants were asked to take off the shoes and any other belongings that could possibly add extra weight when they were weighed. Heights and weights were evaluated according to body mass index (BMI). Also the waist circumference was measured at the level of the iliac processes and the umbilicus with a soft tape measure to estimate abdominal obesity. Blood pressure for each subject was measured twice in the sitting position with an interval of 15 minutes between the measurements, by means of standard sphygmomanometers of appropriate width, after a rest period for 30 minutes. Those taking antihypertensive therapy were considered to have known hypertension [11].

The definitions of the relevant diseases or conditions were as follows: obesity, body mass index (BMI) ≥25 Kg/m^2^; hypercholesterolemia, cholesterol ≥200 mg/dL; high BUN, BUN ≥20 mg/dL; high creatinine, creatinine ≥1.4 mg/dL; and hyperuricemia, uric acid ≥7 mg/dL in men and ≥6 mg/dL in women. Serum ALT or AST levels ≥40 U/L were classified as elevated [4]. Metabolic syndrome (MetS) was diagnosed using the Adult Treatment Panel III (ATP III) criteria, according to the presence of at least 3 of the following 5 risk factors: (1) central obesity (waist circumference ≥90 cm in Asian men and ≥80 cm in Asian women); (2) decreased HDL-C: fasting HDL-C <40 mg/dL or drug treatment for reduced HDL-C; (3) elevated blood pressure: systolic ≥130 mm Hg and/or diastolic ≥85 mm Hg or antihypertensive drug treatment in a patient with a history of hypertension; (4) hypertriglyceridemia: fasting plasma triglycerides ≥150 mg/dL or drug treatment for elevated triglycerides; and (5) hyperglycemia: fasting glucose level ≥100 mg/dL or drug treatment for elevated glucose [12].

### 2.2. Ultrasound Examination and Diagnosis

GSD was diagnosed by a panel of radiologists using real-time ultrasound (TOSHIBA Nemio SSA-550A, Japan). The abdominal regions were examined after the patients had fasted for at least 8 hours, and GSD was identified based on the presence of movable hyperechoic foci with acoustic shadows. GSD was classified as single GSD, multiple GSD, or cholecystectomy, excluding gallbladder polyps. Any type of GSD was considered a case in the study population [4].

### 2.3. Measurements of Interobserver and Intraobserver Reliability

To ensure consistent GSD diagnosis between the 2 specialists, the weighted Kappa statistic was used to assess the agreement of interobserver reliability. A pilot study was performed using 50 randomly selected nonstudy participants and included 4 states of GSD. For interobserver reliability, the weighted Kappa value for the diagnosis of GSD between the 2 specialists was 0.78 (95% confidence interval (CI): 0.70–0.87). For intraobserver reliability, the weighted Kappa value for the diagnosis of GSD was 0.80 (95% CI: 0.75–0.87) for one radiologist and 0.82 (95% CI: 0.74–0.91) for the other radiologist.

### 2.4. Statistical Analysis

Statistical analysis was performed using SAS for Windows (SAS version 9.1; SAS Institute Inc., Cary, NC, USA). Crude and adjusted odds ratios (ORs) (adjusted for sex and age) were estimated, and 95% CIs were applied. Multiple logistic regression was also performed to investigate the independence of the factors associated with the prevalence of GSD. A *P*  value < .05 was considered to represent a statistically significant difference between 2 test populations.

## 3. Results

As shown in Table 1, the overall prevalence of GSD in the study participants was 13.2% (860/6511). The prevalence of GSD was significantly higher in women (14.8%) than in men (12.2%) (for the chi-square test, *P* = .003). According to a Cochran-Armitage trend test, the prevalence of each type of GSD increased with age (*P* < .0001). The participants aged ≥85 years (82/323 = 25.4%) were associated with a more than 2-fold higher risk of GSD compared with the participants aged 60–64 years (225/2140 = 10.5%).

Table 2 presents the crude and adjusted ORs for the associations among certain relevant associated risk factors and GSD prevalence. Compared with the non-GSD participants, in the participants with GSD, female sex (OR = 1.25, 95% CI: 1.08–1.45) and older age (65–74 y versus 60–64 y, OR = 1.25, 95% CI: 1.04–1.50; 75–84 y versus 60–64 y, OR = 1.49, 95% CI: 1.23–1.81; ≥85 y versus 60–64 y, OR = 2.90, 95% CI: 2.18–3.86) were associated with a higher prevalence of obesity (yes versus no, adjusted OR = 1.21, 95% CI: 1.02–1.45), central obesity (yes versus no, adjusted OR = 1.93, 95% CI: 1.64–2.20), hyperglycemia (yes versus no, adjusted OR = 1.37, 95% CI: 1.09–1.66), and MetS (one or 2 metabolic factors versus none, adjusted OR = 1.47, 95% CI: 1.20–1.75; ≥3 metabolic factors versus none, adjusted OR = 1.82, 95% CI: 1.57–2.10), after adjustment for sex and age.

We evaluated the effects of independent associated risk factors for GSD by using a multiple logistic regression model. As shown in Table 3, after adjustment for confounding factors, sex (female versus male, OR = 1.12, 95% CI: 1.03–1.28), age (65–74 y versus 60–64 y, OR = 1.14, 95% CI: 1.02–1.32; 75–84 y versus 60–64 y, OR = 1.22, 95% CI: 1.04–1.41; ≥85 y versus 60–64 y, OR = 2.00, 95% CI: 1.49–2.55), and MetS (one or 2 metabolic factors versus none, OR = 1.47, 95% CI: 1.20–1.75; ≥3 metabolic factors versus none, OR = 1.82, 95% CI: 1.57–2.10) were significantly associated with GSD. Table 3 also lists the results of the multiple logistic regression analysis stratified by sex. Our results indicated that obesity (OR = 1.26, 95% CI: 1.09–1.44) and metabolic factors (one or 2 versus none, OR = 1.48, 95% CI: 1.08–1.76) were significantly associated with GSD in women but not in men.

## 4. Discussion

### 4.1. Prevalence of and Cardiovascular Factors Associated with the Development of GSD

Taiwan has experienced rapid economic development and industrialization, accompanied by changes in traditional diets and increasingly sedentary lifestyles. One of the crucial benefits of early screening for GSD by using ultrasonography is the detection of asymptomatic cases, which can enable the early treatment of GSD and the prevention of serious outcomes such as acute GSD pancreatitis and gallbladder cancer [8, 13]. Few studies have reported the prevalence and possible etiology of GSD in the elderly agricultural and fishing population of Taiwan. Our findings indicate that in this population the prevalence of GSD is higher in women than in men. Although sex as a risk factor for cholelithiasis remains controversial, previous epidemiologic studies have identified higher GSD prevalence in women than in men in Western countries, with estrogen considered the cause of the sex differences [2, 14]. In addition, healthy work effect may affect the correct estimation of prevalent GSD based on voluntarily admitted a physical check-up (self-selection bias).

In this study, we applied the methods for GSD assessment used in previous studies [4, 8], observing a higher prevalence of GSD in an elderly occupational population than that in younger people or the general population. Our results were consistent with those from previous studies conducted in Western countries and other regions of Asia, in which older age was a significant risk factor for GSD [2, 8, 15, 16]. A study on senior citizens in Taiwan similarly demonstrated that age >60 years was the major risk factor for the development of GSD [17]. Long-term exposure to associated risk factors, such as type 2 diabetes, could account for the increased likelihood of GSD development in elderly people [18]. Chronic environmental factors might also contribute to the effects of aging and cause cholelithiasis [18, 19]. In this study, we applied a Cochran-Armitage trend test to confirm that age was associated with the prevalence of GSD. Our results indicated that the pathogenesis of GSD is multifactorial and is likely to develop from complex interactions among several genetic and environmental factors that exert increasing influence with increasing age [8, 18].

Patients with MetS (also known as cardiometabolic syndrome or insulin resistance syndrome) are associated with higher risk of cardiovascular disease irrespective of previous history of cardiovascular events [20]. Previous studies reported that MetS as an insulin resistance phenotype is associated with increased prevalence of GSD [2, 21]. In our analyses, MetS was strongly associated with GSD after we adjusted for confounding factors. This result suggests that the presence of MetS could provide an indicator for the development of GSD. In this study, we classified MetS into 3 categories: no metabolic factors, one or 2 metabolic factors, and ≥3 metabolic factors. By fitting data using a logistic regression model, we estimated the ORs for MetS as 1.33 (95% CI: 1.10–1.54) in the one or 2 metabolic factors group and 1.69 (95% CI: 1.42–1.97) in the ≥3 metabolic factors group, compared with the no metabolic factors group. This finding suggests that the onset of GSD might occur after the development of metabolic disorder, indicating that clinicians should promote health measures for the prevention or early detection of GSD.

Obesity is a major risk factor for GSD development because it is associated with increased hepatic secretion of cholesterol [8, 18]. The underlying mechanism for increased risk of GSD in patients with obesity could be increased bile saturation, resulting from an increase in the biliary secretion of cholesterol. Increased biliary secretion of cholesterol is likely to depend on the high rate of synthesis of cholesterol in obese people [8, 21]. In our study population, we observed that obesity was significantly associated with GSD in women but not in men. In previous studies, men with GSD and high BMI have tended to be associated with other indices of obesity such as slimming treatment and skinfold thickness [8, 22]. Men with GSD can experience loss of muscle bulk and, therefore, tend to gain less weight during adult life than non-GSD men do, despite having a higher proportion of abdominal fat. Men with GSD might lose a higher proportion of lean body mass compared with non-GSD men [22].

### 4.2. Perceived Limitations

One of the major limitations of this study was the evaluation of GSD prevalence and GSD-related risk factors through the screening of an elderly population residing in a single area. However, our study results retained sufficient statistical power for evaluating any sex differences among the various risk factors for GSD because of the considerably large sample size. Second, we evaluated only elderly participants, who might have characteristics that differ from those of the general population. However, this subpopulation is known to be more susceptible to GSD compared with other populations of Taiwan. Third, nonrespondents who did not return for physical examination may have more prevalent GSD or cholecystectomy and the prevalence of GSD may be underestimated. Fourth, we did not evaluate the postcholecystectomy syndrome in this study. This syndrome not only can represent either the continuation of symptoms thought to be caused by the gallbladder or the development of new symptoms normally attributed to the gallbladder but also includes the development of symptoms caused by removal of the gallbladder [23]. Should asymptomatic gallbladder stones be treated with cholecystectomy? This has important clinical implications, not least from a cost-benefit aspect. Postcholecystectomy syndrome may cause suffering and is costly in health care systems. Finally, we conducted measurements at a single time point, which might not reflect long-term exposure to the demographic or biochemical factors related to GSD. Additional prospective longitudinal analogous studies are warranted to verify our cross-sectional findings.

## 5. Conclusion

Our study results indicate that female sex, older age, and MetS are associated with higher prevalence of GSD in the elderly occupational population of Taiwan. In addition, we observed several sex-related differences in the study population. Further investigation is required to elucidate the temporal sequence of events leading to GSD development and to determine the underlying mechanisms of the sex-related differences in GSD prevalence in the elderly population.

## Figures and Tables

**Table 1:** The gender and age specific prevalence of gallstone disease among elderly agricultural and fishing screened subjects (*n* = 6,511).

Variable	Gallstone disease								
	Total	Subtotal		Single stone		Multiple stones		Cholecystectomy	
	Screened	Prevalence		Prevalence		Prevalence		Prevalence	
	Number	Number	(%)	Number	(%)	Number	(%)	Number	(%)
Sex									
Male	3,971	484	12.2	206	5.2	171	4.3	107	2.7
Female	2,540	376	14.8	153	6.0	157	6.2	66	2.6
*P* value for *χ* ^2^-test	0.003								
Age									
60–64	2,140	225	10.5	94	4.4	93	4.3	38	1.8
65–74	2,440	313	12.8	130	5.3	125	5.1	58	2.4
75–84	1,608	240	14.9	100	6.2	81	5.0	59	3.7
85+	323	82	25.4	35	10.8	29	11.1	18	5.5
*P* value for Cochran-Armitage trend test	<0.0001								

Total	6,511	860	13.2	359	5.5	328	5.0	173	2.7

**Table 2:** Univariate analysis of associated clinical factors for gallstone disease among elderly agricultural and fishing screened subjects (*n* = 6,511).

	Gallstone disease		Crude OR (95% CI)	Adjusted OR* (95% CI)
	Yes (*N* = 860)	No (*N* = 5651)		
Sex				
Male	484	3487	1.00	—
Female	376	2164	1.25 (1.08–1.45)	
Age (years)				
60–64	225	1915	1.00	—
65–74	313	2127	1.25 (1.04–1.50)	—
75–84	240	1368	1.49 (1.23–1.81)	—
≥85	82	241	2.90 (2.18–3.86)	—
Obesity				
No	335	2856	1.00	1.00
Yes	525	2795	1.60 (1.38–1.85)	1.21 (1.02–1.45)
Elevated blood pressure				
No	531	3953	1.00	1.00
Yes	329	1698	2.90 (2.18–3.86)	1.42 (0.95–1.88)
Central obesity				
No	512	4377	1.00	1.00
Yes	348	1274	2.34 (2.01–2.71)	1.93 (1.64–2.20)
Hyperglycemia				
No	656	4684	1.00	1.00
Yes	204	967	1.51 (1.27–1.79)	1.37 (1.09–1.66)
Hypercholesterolemia				
No	505	3649	1.00	1.00
Yes	355	2002	1.33 (1.15–1.54)	1.09 (0.81–1.38)
Hypertriglyceridemia				
No	542	3694	1.00	1.00
Yes	318	1957	1.61 (1.03–2.52)	1.54 (0.97–2.46)
Low HDL-C				
No	609	3474	1.00	1.00
Yes	251	2177	0.66 (0.56–0.77)	0.78 (0.55–1.03)
High BUN				
No	704	4809	1.00	1.00
Yes	156	842	1.27 (1.05–1.53)	1.11 (0.94–1.29)
High creatinine				
No	779	5205	1.00	1.00
Yes	81	446	1.21 (0.95–1.56)	1.04 (0.83–1.27)
Hyperuricemia				
No	501	3677	1.00	1.00
Yes	359	1974	1.34 (1.15–1.55)	1.18 (0.82–1.66)
Higher AST				
No	734	5663	1.00	1.00
Yes	127	848	1.16 (0.94–1.41)	1.03 (0.87–1.21)
Higher ALT				
No	716	4785	1.00	1.00
Yes	144	866	1.11 (0.92–1.35)	1.01 (0.90–1.13)
Metabolic syndrome				
None	103	1121	1.00	1.00
One or two	385	2532	1.66 (1.32–2.08)	1.47 (1.20–1.75)
Three or more	372	1998	2.03 (1.61–2.55)	1.82 (1.57–2.10)

*Adjustment for gender and age.

**Table 3:** Multiple logistic regression of association between clinical factors and gallstone disease among elderly agricultural and fishing screened subjects (*n* = 6,511).

Valuable	Gallstone disease (yes versus no)					
	Male		Female		Total	
	OR	95% CI	OR	95% CI	OR	95% CI
Sex (female versus male)	—	—	—	—	1.12	(1.03–1.28)
Age (yrs)						
(65–74 yrs versus 60–64 yrs)	1.09	(1.00–1.20)	1.27	(1.11–1.39)	1.14	(1.02–1.32)
(75–84 yrs versus 60–64 yrs)	1.16	(1.03–1.30)	1.40	(1.18–1.63)	1.22	(1.04–1.41)
(≥85 yrs versus 60–64 yrs)	1.69	(1.33–2.08)	2.14	(1.77–2.52)	2.00	(1.49–2.55)
Obesity (yes versus no)	1.01	(0.86–1.17)	1.26	(1.09–1.44)	1.09	(0.95–1.24)
Metabolic syndrome						
(One or two versus none)	1.13	(0.96–1.40)	1.48	(1.08–1.76)	1.33	(1.10–1.54)
(Three or more versus none)	1.52	(1.29–1.81)	2.01	(1.72–2.30)	1.69	(1.42–1.97)
